# Breaking bad IMRT QA practice

**DOI:** 10.1120/jacmp.v16i3.5242

**Published:** 2015-05-08

**Authors:** Strahinja Stojadinovic, Luo Ouyang, Xuejun Gu, Arnold Pompoš, Qinan Bao, Timothy D. Solberg

**Affiliations:** ^1^ Department of Radiation Oncology University of Texas Southwestern Medical Center Dallas TX USA; ^2^ Department of Radiation Oncology University of Pennsylvania Philadelphia PA USA

**Keywords:** gamma index method, global and local dose difference criteria, divide and conquer gamma method, IMRT commissioning, AAPM Task Group 119, AAPM Task Group 53, ESTRO booklet No.7

## Abstract

Agreement between planned and delivered dose distributions for patient‐specific quality assurance in routine clinical practice is predominantly assessed utilizing the gamma index method. Several reports, however, fundamentally question current IMRT QA practice due to poor sensitivity and specificity of the standard gamma index implementation. An alternative is to employ dose volume histogram (DVH)‐based metrics. An analysis based on the AAPM TG 53 and ESTRO booklet No.7 recommendations for QA of treatment planning systems reveals deficiencies in the current “state of the art” IMRT QA, no matter which metric is selected. The set of IMRT benchmark plans were planned, delivered, and analyzed by following guidance of the AAPM TG 119 report. The recommended point dose and planar dose measurements were obtained using a PinPoint ionization chamber, EDR2 radiographic film, and a 2D ionization chamber array. Gamma index criteria {3%(global),3 mm} and {3%(global),3 mm} were used to assess the agreement between calculated and delivered planar dose distributions. Next, the AAPM TG 53 and ESTRO booklet No.7 recommendations were followed by dividing dose distributions into four distinct regions: the high‐dose (HD) or umbra region, the high‐gradient (HG) or penumbra region, the medium‐dose (MD) region, and the low‐dose (LD) region. A different gamma passing criteria was defined for each region, i.e., a “divide and conquer” (D&C) gamma method was utilized. The D&C gamma analysis was subsequently tested on 50 datasets of previously treated patients. Measured point dose and planar dose distributions compared favorably with TG 119 benchmark data. For all complex tests, the percentage of points passing the conventional {3%(global),3 mm} gamma criteria was 97.2%±3.2% and 95.7%±1.2% for film and 2D ionization chamber array, respectively. By dividing 2D ionization chamber array dose measurements into regions and applying 3 mm isodose point distance and variable local point dose difference criteria of 7%, 15%, 25%, and 40% for HD, HG, MD, and LD regions, respectively, a 93.4%±2.3% gamma passing rate was obtained. Identical criteria applied using the D&C gamma technique on 50 clinical treatment plans resulted in a 97.9%±2.3% gamma passing score. Based on the TG 119 standard, meeting or exceeding the benchmark results would indicate an exemplary IMRT QA program. In contrast to TG 119 analysis, a different scrutiny on the same set of data, which follows the AAPM TG 53 and ESTRO booklet No.7 guidelines, reveals a much poorer agreement between calculated and measured dose distributions with large local point dose differences within different dose regions. This observation may challenge the conventional wisdom that an IMRT QA program is producing acceptable results.

PACS number: 87.55.Qr

## I. INTRODUCTION

The gamma index evaluation method was introduced in a seminal work by Low et al.^(1)^ in 1998. This method enabled comparison of dose distributions in a quantitative manner by calculating the gamma index, the minimum distance in the normalized dose‐distance space. The normalization is performed by dividing every dose and spatial coordinate by user selected dose difference (ΔD cGy) and isodose point distance (Δd mm) criteria respectively, resulting in unit‐less quantities which can be evaluated simultaneously. The original gamma evaluation method has been refined to provide more efficient calculations in terms of speed and accuracy^2^, ^3^, ^4^, ^5^, ^6^ and to extend and improve the capabilities of the concept.^7^, ^8^, ^9^, ^10^, ^11^, ^12^, ^13^, ^14^, ^15^

The AAPM TG 119 report^(16)^ describes benchmark commissioning tests provided to assess the overall accuracy of planning and delivery of IMRT treatments. The report also presents multi‐institutional baseline expectation values based on gamma index analysis using 3 mm isodose point distance and 3% dose difference acceptance criteria. The 3% dose difference per TG 119 is relative to the point of maximum dose. Hence, the dose denominator for gamma calculations is the percent value of the maximum measurement point, i.e., a global normalization value, not the percent value of the local dose.

Recent publications revealed practical problems with important clinical implications when performing patient‐specific quality assurance based on the gamma index method. A number of peer reviewed publications demonstrated that patient dose errors have a weak correlation with gamma passing rates for IMRT QA,^17^, ^18^ that single field IMRT measurements can be insensitive to dosimetric inaccuracies of the overall plan,^19^, ^20^ that there is lack of correlation between global gamma indices and clinical DVH metrics,^21^, ^22^ and that the gamma index method does not guarantee the absence of clinically significant dose deviations.^23^, ^24^ In addition, published opinions raised concern about poor sensitivity and specificity of the standard gamma algorithm^(25)^ and pointed out significant limitations of the AAPM TG 119 report.^26^, ^27^ This is closely related to the Radiological Physics Center (RPC) anthropomorphic head and neck phantom credentialing results.^(28)^ Based on the RPC standard, merely 82% of institutions passed the end‐to‐end test with 7% dose difference and 4 mm isodose point distance criteria, and only 69% of institutions passed a more stringent 5% dose criterion.^(28)^ These reports fundamentally question clinical utilization of the gamma index method based on a global dose difference.

The AAPM TG 53 report^(29)^ and ESTRO booklet No.7,^(30)^ based on work by Van Dyk et al.^(31)^ and Venselaar et al.,^(32)^ proposed a method for characterization of the accuracy of dose calculations and corresponding measurements. For analysis of agreement between calculations and measurements, a dose distribution is segmented into significant regions: central axis, inner beam, penumbra, outer beam, and buildup region. Each region is accompanied with suggested acceptability criteria for various beam configurations. The key recommendation is that “these regions should be analyzed separately, so that reasonable characterization of the agreement between calculations and data can be performed without combining the regions of large dose gradients with those which have small gradients”. In retrospect, it seems that this guidance was clearly overlooked by AAPM TG 119. As a consequence, the overarching {3%(global),3 mm} gamma index criteria was recommended, however, this indeed signifies a bad metric, as corroborated with experimental findings discussed above.^17^, ^18^, ^19^, ^20^, ^21^, ^22^, ^23^, ^24^ Moreover, these regions have different levels of calculated dose uncertainty relative to measurements for all dose calculation algorithms. This suggests that even {3%(global),3 mm} gamma index criteria, or for that matter any uniform local dose difference criterion across all regions, would not represent a good metric for evaluating dose distributions. It is no surprise that several authors have recommended replacing the gamma index method with a DVH‐based^18^, ^24^ patient‐specific IMRT QA analysis. In fact, with a consensus guidance document on generally accepted criteria for evaluating DVH‐based metrics, this avenue could become a new standard for patient‐specific QA.

The gamma index approach, however, can be significantly improved by applying a “divide and conquer” (D&C, in Latin: *divide et impera*) method, which in essence follows AAPM TG 53^(29)^ and ESTRO booklet No.7^(30)^ by segmenting dose distributions into regions^(31)^ and analyzing each region separately. The “divide and conquer” approach is founded on well‐known concepts of confidence limits and action levels for various dose regions proposed by Venselaar et al.^(32)^ and refined by Palta et al.^(33)^ and adapted to the gamma index method of Low et al.^(1)^ The premise of this work is that the gamma index method overall is a remarkable concept and exceptionally useful tool, and that the deficiencies described in an increasing body of publications are due to the implementation rather than the method itself. This is an important distinction, as the implementation of the method is intimately linked to the selection of acceptance criteria, which is what ultimately defines the metric for evaluation.

In this study, the TG 119 benchmark IMRT tests were analyzed using the “divide and conquer” gamma index implementation. In light of the new metric, the findings may challenge the existing “state of the art” IMRT QA practice.

## II. MATERIALS AND METHODS

### A. Deficiencies of the global gamma index method

The gamma (γ) index represents the minimum Euclidean distance in the normalized dose‐distance space:^(1)^

(1) $$\gamma =\text{min}\{\Gamma ({\vec{r}}_{eval},{\vec{r}}_{ref})\},\forall \{{\vec{r}}_{eval}\}$$

(2) $$\text{where}\;\Gamma ({\vec{r}}_{eval},{\vec{r}}_{ref})=\sqrt{\frac{{\left|{\vec{r}}_{eval},{\vec{r}}_{ref}\right|}^{2}}{\Delta d^{2}}+\frac{{\left(D_{eval}({\vec{r}}_{eval})-D_{ref}({\vec{r}}_{ref})\right)}^{2}}{\Delta D^{2}}}$$

The γ function is the minimum of generalized Γ function computed for arbitrary isodose point distance Δd and dose difference ΔD values, for all evaluated ${\vec{r}}_{eval}$ and reference positions ${\vec{r}}_{ref}$, with corresponding evaluated $D_{eval}({\vec{r}}_{ref})$ and reference doses $D_{ref}({\vec{r}}_{ref})$. Note that in the literature Δd is universally called distance‐to‐agreement (DTA). The label DTA for Δd is in fact quite ambiguous as it implies agreement where there may be none. It is more accurate to call Δd the isodose point distance, bearing in mind that this is exactly the meaning of Δd in Eq. (2). The Δd value has been specified as either percent dose relative to a global normalization point or percent value with respect to a dose at the local point.

Choosing any global dose difference Δd value is the predominant deficiency of the gamma index method. The following three arguments, each one self‐sufficient, concisely describe the deficits of the standard (global) gamma implementation:

1. TG 119 recommends using the point of maximum dose $D_{\text{max}}$ as global normalization value. Hence, the dose denominator Δd for gamma calculations is the percent value of the maximum measurement point which, by definition, guarantees the best possible gamma passing rate. Clearly the goal should be to faithfully quantify dose distributions agreements and not to have the highest passing rate invoked by a biased convenience which hides dose discrepancies.
2. In general, for two given dose distributions $D_{\text{measured}}$ and $D_{\text{calculated}}$, at every point their relative difference is defined as $(D_{measured}-D_{calculated})/D_{calculated}$. The second term in Eq. (2), $(D_{eval}({\vec{r}}_{eval})-D_{ref}({\vec{r}}_{ref}))/\Delta D$, is interpreted as relative dose difference between two points in evaluated and reference dose distributions. When Δd is not the local point dose, or in a vicinity of that point, then the whole term can neither be considered nor does it represent a relative dose difference. To illustrate these circumstances, picture currently the world's tallest building, Burj Khalifa in Dubai (height 828 m), and two skyscrapers from Chicago, the Willis Tower (height 442 m) and the Trump International Hotel and Tower (height 423 m). The height difference between the Willis and Trump Towers could be assessed using an absurd metric depicted by the following question: “Is the relative height difference between the Willis Tower and the Trump Tower within 3% compared to Burj Khalifa height”. The absurd answer would be: yes, since (442 m−423 m)/(0.03⋅828 m)<1.
3. The intrinsic quandary with using a global normalization value ΔD for the gamma index method is that there are an infinite number of possible points from which to choose. The two most commonly chosen points are the isocenter and the point of maximum dose. Aside from convenience, however, these two points are no more relevant or important than any other point. Moreover, a point which provides the lowest gamma index passing rate is as good dose normalization choice as any other point. In reality, the selection of any global dose normalization point only provides an answer in a relative sense, i.e., relative to that point. This practice produces biased results and offers irrelevant skewed perspective from one point in a world of infinite choices.

### B. Deficiencies of the local gamma index method

The infinite number of solutions for a global gamma index comparison is easily resolved by requiring ΔD to be a percent value of the dose at the local point in Eq. (2). Note that in this scenario, a local dose difference approach provides just one solution, i.e., a unique answer to the question of how well two isodose distributions agree. Moreover, the second term in Eq. (2), $(D_{eval}({\vec{r}}_{eval})-D_{ref}({\vec{r}}_{ref}))/\Delta D$ now properly represents a relative dose difference between two points in the evaluated and reference dose distributions.

Based on the works of Van Dyk et al.^(31)^ and Venselaar et al.,^(32)^ TG 53^(29)^ and ESTRO^(30)^ recommend an alternate technique for analyzing calculated and delivered dose distributions in which dose distributions are partitioned into meaningful regions and analyzed separately. Since the corresponding dose uncertainties are different for each region, however, the local gamma index method utilizing uniform local dose difference criterion across all regions is not a good metric. The deficiencies of such implementation have been corroborated in several published works.^17^, ^20^, ^21^, ^24^

### C. Requisite of the “divide and conquer” gamma index method

In this study, the deficiencies of the uniform local dose difference approach are addressed through the “divide and conquer” gamma index method. Dose distributions are divided in four distinct regions and for each region different gamma criteria are defined.

The four regions of any dose distribution are: the high‐dose (HD) or umbra region, the high‐gradient (HG) or penumbra region, the medium‐dose (MD) region, and the low‐dose (LD) region. The definition of each region is given in Table 1. The HD region includes isodose points equal to or greater than 90% of the maximum planar dose $D_{\text{max}}$. The HG region is a union of isodose levels encompassing points with 90% to 50% of $D_{\text{max}}$ ^(32)^ and points which exhibit more than 3% dose variation within a millimeter.^(31)^ The MD region contains isodose points within 50% and 20% of $D_{\text{max}}$. The LD region surrounds isodose points between 20% and 10% of $D_{\text{max}}$, setting 0.1 · D^max^ as a threshold isodose level.

**Table 1:** Definition of four distinct regions of an arbitrary dose distribution.

*Region*	*Selection Criteria*
Umbra: High Dose (HD)	$HD(\vec{r})\in \left\{0.9\cdot D_{\text{max}}\leq D(\vec{r})\leq D_{\text{max}}\right\},\forall \{\vec{r}\}$
Penumbra: High Gradient (HG)	$HG(\vec{r})\in \left\{0.5\cdot D_{\text{max}}\leq D(\vec{r})<0.9\cdot D_{\text{max}}\right\}\cup \left\{\Delta D(\vec{r})\geq 3\%/mm\right\},\forall \{\vec{r}\}$
Medium Dose (MD)	$MD(\vec{r})\in \left\{0.2\cdot D_{\text{max}}\leq D(\vec{r})<0.5\cdot D_{\text{max}}\right\},\forall \{\vec{r}\}$
Low Dose (LD)	$LD(\vec{r})\in \left\{0.1\cdot D_{\text{max}}\leq D(\vec{r})<0.2\cdot D_{\text{max}}\right\},\forall \{\vec{r}\}$

### D. Treatment planning

The TG 119 test suite structures were downloaded in DICOM format from the AAPM website and applied to the CT scan of a Solid Water phantom. The phantom consisted of Solid Water slabs with a cross‐sectional area of $30\times 30\%{\text{cm}}^{2}$ and a total thickness of 22 cm, with a centrally located pinpoint ionization chamber 11 cm below the anterior surface. The phantom was scanned using a Brilliance CT Big Bore (Phillips Healthcare, Andover, MA) and imported to Pinnacle (Philips Medical Systems, Inc., Fitchburg, WI) for treatment planning. IMRT tests with increasing complexity were optimized using TG 119 specifications including the number of beams and beam arrangement. The TG 119 test I5, called “Hard C‐Shape” was excluded as meeting the planning constraints is not feasible.^16^, ^34^ The results presented in the TG 119 report were exclusively for 6 MV photons, whereas the data presented in this study also include additional evaluations for 18 MV photons. All dose calculations were performed with heterogeneity corrections using the collapsed cone convolution dose algorithm. The optimization was performed utilizing the direct machine parameter optimization (DMPO) algorithm with the following clinical IMRT parameters: maximum 15 segments per beam, 2 cm^2^ minimum segment area, minimum 3 MUs per segment, 2 cm minimum overlap distance for beam splitting, 5 mm leaf/field edge overlap and a 2^3^ mm^3^ calculation grid. Following each optimization, the plans were recalculated on a 2D ionization chamber array (MatriXX Evolution, IBA Dosimetry America, Bartlett, TN) in the MULTICube configuration. The dimensions of MatriXX / MULTICube are $31.4(L)\times 34(W)\times 22(H)\;{\text{cm}}^{3}$ which closely resembles the Solid Water phantom dimensions of $30\times 30\times 22\;{\text{cm}}^{3}$. Each plan was transferred to a record and verify system (MOSAIQ, Elekta – IMPAC Medical Systems, Inc., Sunnyvale, CA) for delivery on a Varian 21EX linac using 4D ITC with 120‐leaf Millennium MLC (Varian Medical Systems, Inc., Palo Alto, CA).

### E. Measurements

The absorbed dose measurements were recorded at locations specified by TG 119. A PinPoint ionization chamber PTW 31014 (PTW – New York Corporation, Hicksville, NY), with a sensitive volume of 0.015 cm^3^, was used for point dose measurements. The point dose differences between measured and planned values, per TG 119 recommendations, are expressed as a percentage ratio relative to the prescribed dose. The planar dose distributions were recorded in a coronal plane, using Kodak extended dose range EDR2 film (Carestream Health, Inc., Rochester, NY) and using the MatriXX in the MULTICube configuration. In view of the fact that the measurements were performed over multiple days, the effects of linac output variation were accounted for by measuring output in a water phantom following the AAPM's TG 51 protocol for clinical reference dosimetry^(35)^ for each irradiation session.

### F. Data analysis

The Kodak EDR2 film planar dose distributions were analyzed using FILMQA (3cognition LLC, Wayne, NJ). The MatriXX planar dose distributions were analyzed utilizing OmniPro I'mRT (IBA Dosimetry America). Per TG 119 recommendations, planned and measured data comparisons are presented in the form of a confidence limit CL defined as:

(3) CL=|100−Mean|+1.96⋅σ

where *Mean* is the average percentage of points passing the gamma criteria and σ is the standard deviation.

The analysis of composite planned and delivered dose distributions was performed in four steps:

1. First, data were evaluated strictly per TG 119 recommendations, i.e., utilizing {3%(global),3 mm} gamma index criteria. This means that the dose denominator ΔD for gamma calculations was 3% of the value of the maximum dose $D_{\text{max}}$, i.e., a global normalization value, not the percent dose value at the local point.
2. Second, data were examined in light of {3%(global),3 mm} gamma index criteria. In this case the dose denominator ΔD for gamma calculations was 3% of the value of the local dose point.
3. Third, data were analyzed using the “divide and conquer” gamma index method. Dose distributions were divided in the four regions, as described in Materials & Methods section C., and each region had different local gamma index criteria.
4. Finally, an analysis of variance (ANOVA) test was utilized for statistical analysis of the average values of the resulting gamma passing rates.

The crucial question for the “divide and conquer” gamma index method is to determine the appropriate gamma criteria for each region. Rather than selecting arbitrarily, criteria were determined iteratively using a GPU‐based fast gamma index calculation algorithm,^(6)^ with the region specific local dose denominator used as a free search parameter for greater than 90% combined gamma passing score in all regions. Using an isodose point distance Δd larger than 3 mm would imply accepting a geometric miss, thus the Δd criterion was fixed at 3 mm for each region.

As a final point, the “divide and conquer” gamma index method was applied in a retrospective analysis of 50 clinically treated patients. The IMRT plans were randomly chosen between the entire dosimetry group. The intent was to sample dissimilar IMRT dose patterns planned by different physicians and dosimetrists. The plans represented a variety of sites including brain (10 plans), head and neck (12 plans), lung (8 plans), pelvis (8 plans), spine (2 plans), and prostate (10 plans). The brain and pelvis plans utilized 6, 10 or 18 MV photons, all H&N and prostate cases employed 6 MV and 10 MV photons, respectively, while lung and spine cases used either 6 or 10 MV photons. Clinical IMRT QA MatriXX isodose distributions for these 50 patients, all of which previously demonstrated 90% or better gamma index passing rates using {3%(global),3 mm} criteria were reanalyzed to determine which variable local point dose difference criteria would yield a set goal of 90% gamma index passing scores.

## III. RESULTS

### A. Treatment planning results

The planning goals^(16)^ for TG 119 IMRT benchmark tests including target coverage and constraints for critical structures were all met.

### B. Ionization chamber results

A comparison with the TG 119 benchmark data indicates that ionization chamber dose measurements met or exceeded individual results obtained by TG 119 participating institutions. At the same time, the average point dose measured values were in excellent agreement with published TG 119 ionization chamber measurements. In the high‐dose region, the average point dose measurements for the ten TG 119 institutions [(Mean±SD); within CL] was $[(-0.002\pm 0.022);{0.045]}_{\text{TG}119}$ compared to $[(0.006\pm 0.015);{0.034]}_{\text{UTSW}}$. Similarly, in the low‐dose region the TG 119 average was $[(0.003\pm 0.022);{0.047]}_{\text{TG}119}$ compared to $[(0.006\pm 0.014);{0.034]}_{\text{UTSW}}$.

### C. Composite radiographic film results

The comparison of planned and delivered coronal dose distributions measured using EDR2 film in a Solid Water phantom are shown in Table 2. The films were analyzed utilizing FILMQA software. The analysis was performed utilizing TG 119 recommended {3%(global),3 mm} gamma criteria, i.e., the dose denominator ΔD for gamma calculations was 3% value of the maximum dose $D_{\text{max}}$. For each plan, the gamma index passing scores, indicating points with gamma ≤ 1.0, are shown in Table 2. On average, the combined film measurements for both 6 and 18 MV photon energies resulted in a 97.2%±3.2% gamma passing score, well within the published TG 119 action level threshold of 88%.

**Table 2:** Results of film planar dose measurements using the point of maximum dose as global normalization point with percentage of points passing the {3%(global),3 mm} gamma criteria.

*Plan*	*Plane*	*6 MV Gamma Passing Rate (%)*	*Plane*	*18 MV Gamma Passing Rate (%)*
I1 Multitarget	Isocenter	98.4	Isocenter	94.4
I2 Mock Prostate	Isocenter	98.3	Isocenter	99.6
	2.5 cm posterior	100.0	2.5 cm posterior	100.0
I3 Mock H&N	Isocenter	97.6	Isocenter	95.6
	4.0 cm posterior	89.3	4.0 cm posterior	98.7
I4 Easy C‐Shape	Isocenter	92.6	Isocenter	99.1
	2.5 cm anterior	99.3	2.5 cm anterior	98.7
	6 MV Mean	96.5	18 MV Mean	98.0
	6 MV SD	4.0	18 MV SD	2.1
	6 MV CL	11.3	18 MV CL	6.2
	Combined (6 & 18 MV) Mean		97.2	
	Combined Standard Deviation		3.2	
	Combined Confidence Limit		9.0	

### D. Composite 2D ionization chamber array results

In addition to film measurements, planar dose distributions were recorded using the MatriXX Evolution in a MULTICUBE configuration. The resulting ionization chamber array measurements were evaluated per TG 119 recommendations using OmniPro I'mRT. Planned and delivered planar dose distributions were analyzed using the {3%(global),3 mm} criteria, with a dose denominator ΔD of 3% of $D_{\text{max}}$ for gamma calculations. For each plan, the passing gamma index percent values, corresponding to points with gamma ≤ 1.0, are reported in Table 3. The combined MatriXX gamma passing score was 95.7%±1.2% for both 6 and 18 MV photon energies. As with the film measurements, the MatriXX 2D ionization chamber array measurements were well within the published TG 119 action level threshold of 88%.

**Table 3:** Results of MatriXX planar dose measurements using the point of maximum dose as global normalization point with percentage of points passing the {3%(global),3 mm} gamma criteria.

*Plan*	*Plane*	*6 MV Gamma Passing Rate (%)*	*Plane*	*18 MV Gamma Passing Rate (%)*
I1 Multitarget	Isocenter	93.6	Isocenter	95.4
I2 Mock Prostate	Isocenter	96.5	Isocenter	96.0
I3 Mock H&N	Isocenter	95.9	Isocenter	96.7
I4 Easy C‐Shape	Isocenter	97.1	Isocenter	94.2
	6 MV Mean	95.8	18 MV Mean	95.6
	6 MV SD	1.6	18 MV SD	1.0
	6 MV CL	7.3	18 MV CL	6.5
	Combined (6 & 18 MV) Mean		95.7	
	Combined Standard Deviation		1.2	
	Combined Confidence Limit		6.8	

### E. Reanalysis of composite 2D ionization chamber array measurements

The preceding analysis demonstrates that the IMRT benchmark results are in line with published peer reviewed baseline data for a well‐commissioned IMRT program.

#### E.1 The local gamma index method results

A GPU‐based fast gamma index calculation algorithm^(6)^ was utilized to reanalyze data with {3%(global),3 mm} criteria and with 10% of $D_{\text{max}}$ set as a threshold isodose level. As a result, a dramatic drop in gamma index passing rates was observed, as shown in Table 4.

**Table 4:** Percentage of points passing the {3%(global),3 mm} gamma criteria for composite MatriXX planar dose measurements.

*Plan*	*Plane*	*6 MV Gamma Passing Rate (%)*	*Plane*	*18 MV Gamma Passing Rate (%)*
I1 Multitarget	Isocenter	28.8	Isocenter	18.7
I2 Mock Prostate	Isocenter	18.5	Isocenter	12.2
I3 Mock H&N	Isocenter	12.9	Isocenter	9.5
I4 Easy C‐Shape	Isocenter	22.6	Isocenter	15.3
	6 MV Mean	20.7	18 MV Mean	13.9
	6 MV SD	6.7	18 MV SD	4.0
	6 MV CL	92.4	18 MV CL	93.8
Overall (6 & 18 MV) Mean		17.3	
	Overall Standard Deviation		6.2	
	Overall Confidence Limit		94.9	

**Figure 1:**
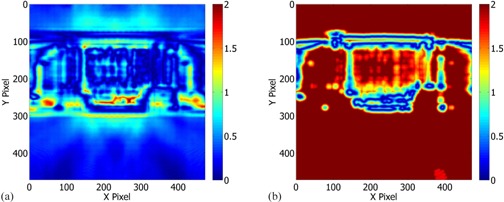
Composite gamma index map for I3 ‐ Mock H&N 6 MV plan: (a) {3%(global),3 mm} gamma criteria resulted in 95.9% passing rate; (b) {3%(global),3 mm} gamma criteria resulted in 12.9% passing rate.

On average, the mean, standard deviation and confidence limit values of [(95.7%±1.2%);6.8%] were reduced drastically to [(17.3%±6.2%);94.9%] after changing from 3% global to 3% local dose difference, respectively. Figure 1(a) shows a gamma index map for the I3 Mock H&N 6 MV plan using {3%(global),3 mm} criteria. Figure 1(b) shows a gamma index map for the identical plan using {3%(global),3 mm} criteria. As indicated in Table 4, the 3% local point dose difference criterion seems to be too stringent for intrinsically complex intensity‐modulated fields, yielding comparisons with very poor gamma index passing rates. The low passing rates also suggest that universal local dose gamma criteria across the entire dose distribution cannot be employed to any further extent.

#### E.2 The “divide and conquer” gamma index method results

**Figure 2:**
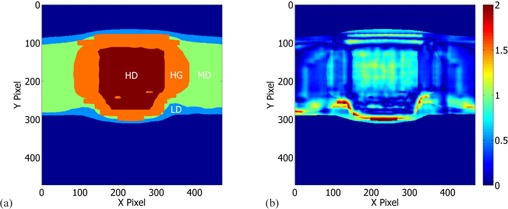
The “divide and conquer” gamma index method. Segmentation of reference isodose distribution for I3 ‐ Mock H&N 6 MV plan into four distinct regions; for each region different gamma passing criteria were defined. (a) Four regions of interest: the high‐dose (HD) or umbra region, the high‐gradient (HG) or penumbra region, the medium‐dose (MD) region, and the low‐dose (LD) region. Dark blue region indicates the excluded 10% threshold dose relative to the maximum planar dose. (b) 94% passing score for the “divide and conquer” gamma index method utilizing the 3 mm isodose point distance and variable local point dose difference criteria of 7%, 15%, 25%, and 40% for HD, HG, MD, and LD regions, respectively.

The planar dose distributions were segmented in four distinct regions, HD, HG, MD, and LD, defined in Table 1 and illustrated in Fig. 2(a). Each region had a variable dose difference criterion, while the 3 mm isodose point distance criterion was fixed for all regions. Next, the variable local point dose difference criterion was iteratively determined such that clinically acceptable, i.e., larger than 90% gamma index passing rates would be obtained. Ultimately, a 93.4%±2.3% average gamma passing score for all plans was obtained, see Table 5. The corresponding iteratively found variable local point dose differences were 7%, 15%, 25%, and 40% for the HD, HG, MD, and LD regions, respectively. Figure 2(b) shows a “divide and conquer” gamma index map for I3 Mock H&N 6 MV plan using variable gamma criteria.

**Table 5:** Percentage of points passing the 3 mm isodose point distance and variable local point dose difference criteria of 7%, 15%, 25%, and 40% for HD, HG, MD, and LD regions, respectively for MatriXX planar dose measurements.

*Plan*	*Plane*	*6 MV Gamma Passing Rate (%)*	*Plane*	*18 MV Gamma Passing Rate (%)*
I1 Multitarget	Isocenter	93.9	Isocenter	95.9
I2 Mock Prostate	Isocenter	91.7	Isocenter	91.9
I3 Mock H&N	Isocenter	94.0	Isocenter	95.0
I4 Easy C‐Shape	Isocenter	95.5	Isocenter	89.3
	6 MV Mean	93.8	18 MV Mean	93.3
	6 MV SD	1.6	18 MV SD	3.0
	6 MV CL	9.3	18 MV CL	12.9
	Overall (6 & 18 MV) Mean		93.4	
	Overall Standard Deviation		2.3	
	Overall Confidence Limit		11.0	

### F. Analysis of clinical data

The “divide and conquer” gamma index concept was subsequently tested on a retrospective set of 50 IMRT treated patients. Each of the 50 IMRT QA MatriXX datasets originally had clinical gamma index passing scores of 90% or better using {3%(global),3 mm} criteria within a region of interest defined as 10% threshold dose relative to the maximum planar dose. For these particular datasets, the results in TG 119 format, for the mean, standard deviation, and confidence limit were [(95.1%±3.1%);10.9%], respectively. After applying the {3% (local),3 mm} gamma criteria, the results dropped to [(56.9%±10.0%);62.7%]. Next, all 50 planar dose distributions were segmented in four regions, as defined in Table 1. The metric for dose comparisons was determined by using fixed 3 mm isodose point distance criterion in conjunction with variable local point dose difference criteria of 7%, 15%, 25%, and 40% for HD, HG, MD, and LD regions, respectively. Following the analysis, the “divide and conquer” gamma index passing rates of [(97.9%±2.3%);6.7%] were achieved.

### G. ANOVA F‐test analysis

A single factor ANOVA F‐test was utilized for statistical analysis of the average values of the resulting gamma passing rates. The ANOVA method compares the data calculated value F with the critical value $F_{\text{critical}}$ determined from the f‐distribution in statistical tables. If $F\geq F_{\text{critical}}$, the null hypothesis is rejected. The null hypothesis is that the mean gamma passing rates in 3, 5 are the same for both TG 119 and “divide and conquer” metrics. For all benchmark plans in 3, 5, the obtained F values are: $F=6.2>F_{\text{critical}}=4.6$. Hence, the null hypothesis is not true and should be rejected. Using the same null hypothesis and repeating the test on 50 patients' datasets, the obtained F values are: $F=26.6>F_{\text{critical}}=3.9$. Therefore, the TG 119 and “divide and conquer” metrics provide statistically significant differences in gamma passing rates, i.e., they paint two different realities about agreement between planned and delivered dose distributions.

## IV. DISCUSSION & CONCLUSIONS

Segmentation of isodose distributions into four distinct regions, as defined in Table 1, and utilization of variable gamma criteria in a local sense for different regions can generate larger than 90% gamma passing scores which indicate clinical acceptability. Dose distributions segmentation into four regions was based on the recommendations of AAPM TG 53 report^(29)^ and ESTRO booklet No.7.^(30)^ Specifically, the regions threshold values were adapted from Van Dyk et al.^(31)^ and Venselaar et al.^(32)^ What are the optimal number of regions and how to define the optimal regions specific threshold values are open questions for further research. The rationale for choosing 90% gamma passing rate as clinically acceptable was based on the TG 119^(16)^ average 88% gamma passing score for composite film measurements. From a clinical perspective, however, it is noteworthy to consider that not all dose regions may be equally essential. This could be taken into account by using variable passing score criteria for various regions. Possibly a consensus gamma passing score goal for each region could be set based on data from multiple institutions, similar to TG 119 approach. In this study, acceptable results were obtained using fixed 3 mm isodose point distance and variable local point dose difference criteria of 7%, 15%, 25%, and 40% for HD, HG, MD, and LD regions, respectively.

The TG 119 and “divide and conquer” methods present two different perspectives about agreement between planned and delivered dose distributions with statistically significant differences in gamma passing rates. Two diametrically opposite conclusions can be derived from results of this work. An exemplary commissioned IMRT QA program is the first conclusion, as all treatment planning tasks, point dose, film, and 2D ionization chamber array dose measurements are in excellent agreement with TG 119 published data. In contrast to TG 119 analysis, a different scrutiny on the same set of data reveals an alarmingly diverse reality. This other perspective has relatively large 7%, 15%, 25%, and 40% local point dose differences for different dose regions. Certainly, the second conclusion would point to deficiencies in an IMRT QA program. An interesting question is whether or not this is truly a deficiency or are these local point dose differences acceptable, i.e., is this the best one can do based on thorough scrutiny of overall accuracy of the IMRT process? Addressing this matter requires a detailed understanding of the uncertainties throughout the treatment planning and delivery processes.

It is quite difficult to accept this unattractive realization based on years of an effortless and soothing {3%(global),3 mm} metric, which offers excellent passing analysis but reveals very little and does not correlate to clinical DVH metrics. However, the findings of the “divide and conquer” method are not as arbitrary as it may seem at first sight. For example, Howel et al.^(36)^ reported an average of 40% local dose difference for 238 out‐of‐field points of measurement for a historic AP/PA mantle plan. For open fields, Bednarz and Xu^(37)^ found that the average local difference between calculated and measured out‐of‐field doses for the 6 and 18 MV beams were 14% and 16%, respectively. For three IMRT plans studied, Huang et al.^(38)^ showed that the out‐of‐field dose was on average underestimated by a commercial treatment planning system by 50%. Furthermore, Joosten et al.^(39)^ considered an uncertainty of 50% in dose estimation acceptable in the context of assessing the risk of secondary cancers, and showed that the peripheral dose between two linacs could differ by up to a factor of 9 for small fields and up to a factor of 10 for wedged fields.

These and other references are part of a large body of publications which reveal large dose uncertainties. Nonetheless, such dose discrepancies are not typically discovered with a {3%(global),3 mm} gamma analysis. To date, the only alternative to the gamma method suggested in the literature is to use DVH‐based metrics. It must not be overlooked that in contrast to DVHs for planning tumor volumes (PTVs), DVHs of organs at risk (OARs) are a result of dose summations of numerous blocked fields, i.e., summations of multiple out‐of‐field doses. This fact raises a significant concern that OAR DVHs are, for the most part, generated by commercial treatment planning systems which have large out‐of‐field dose uncertainties. This, in turn, translates to standard clinical DVH graphs which have very little correlation to reality, yet the quality of patients care is judged by such DVHs. Simply put, both global gamma and DVH‐based analyses are inadequate, as the “divide and conquer” gamma method presented here confirms.

A better paradigm would be to standardize IMRT QA practices by minimizing observed local point dose differences. Several new distinctive developments need to happen to provide meaning with regard to the current IMRT QA standard. First, commissioning of treatment planning systems must include out‐of‐field specific considerations, perhaps to the same level of detail as is now devoted to open fields. This is an opportunity of exploration which has been ignored to a great extent. Second, dose uncertainties should be reported alongside dose for every voxel in all treatment planning systems. More to the point, addition of dose for multiple beams must reflect proper dose uncertainty propagation associated with dose summation. For instance, if dose uncertainty along the central axis is 1%, then for an isocentric four‐field box plan, after summing the uncertainty for each beam in quadrature, a 2% isocenter dose uncertainty should be reported alongside the calculated dose. This is of utmost importance for validation of calculated dose with measurements. Of course, the measurement itself has an intrinsic uncertainty that must also be considered. Therefore, a meaningful comparison between calculation and measurement is only possible when both calculations and measurements agree within their estimated errors, something that is not currently considered in the present IMRT QA paradigm.

## References

[1] Low DA , Harms WB , Mutic S , Purdy JA . A technique for the quantitative evaluation of dose distributions. Med Phys. 1998;25(5):656–61. 9608475 10.1118/1.598248

[2] Wendling M , Zijp LJ , McDermott LN , et al. A fast algorithm for gamma evaluation in 3D. Med Phys. 2007;34(5):1647–54. 17555246 10.1118/1.2721657

[3] Ju T , Simpson T , Deasy JO , Low DA . Geometric interpretation of the gamma dose distribution comparison technique: interpolation‐free calculation. Med Phys. 2008;35(3):879–87. 18404924 10.1118/1.2836952

[4] Chen M , Lu W , Chen Q , Ruchala K , Olivera G . Efficient gamma index calculation using fast Euclidean distance transform. Phys Med Biol. 2009;54(7):2037–47. 19287084 10.1088/0031-9155/54/7/012

[5] Yuan J and Chen W . A gamma dose distribution evaluation technique using the k‐d tree for nearest neighbor searching. Med Phys. 2010;37(9):4868–73. 10.1118/1.3480964 20964204

[6] Gu X , Jia X , Jiang SB . GPU‐based fast gamma index calculation. Phys Med Biol. 2011;56(5):1431–41. 21317484 10.1088/0031-9155/56/5/014 PMC3156145

[7] Depuydt T , Van Esch A , Huyskens DP . A quantitative evaluation of IMRT dose distributions: refinement and clinical assessment of the gamma evaluation. Radiother Oncol. 2002;62(3):309–19. 12175562 10.1016/s0167-8140(01)00497-2

[8] Childress NL and Rosen II . The design and testing of novel clinical parameters for dose comparison. Int J Radiat Oncol Biol Phys. 2003;56(5):1464–79. 12873692 10.1016/s0360-3016(03)00430-9

[9] Bakai A , Alber M , Nusslin F . A revision of the gamma‐evaluation concept for the comparison of dose distributions. Phys Med Biol. 2003;48(21):3543–53. 14653561 10.1088/0031-9155/48/21/006

[10] Low DA and Dempsey JF . Evaluation of the gamma dose distribution comparison method. Med Phys. 2003;30(9):2455–64. 14528967 10.1118/1.1598711

[11] Stock M , Kroupa B , Georg D . Interpretation and evaluation of the gamma index and the gamma index angle for the verification of IMRT hybrid plans. Phys Med Biol. 2005;50(3):399–411. 15773719 10.1088/0031-9155/50/3/001

[12] Jiang SB , Sharp GC , Neicu T , Berbeco RI , Flampouri S , Bortfeld T . On dose distribution comparison. Phys Med Biol. 2006;51(4):759–76. 16467577 10.1088/0031-9155/51/4/001

[13] Spezi E and Lewis DG . Gamma histograms for radiotherapy plan evaluation. Radiother Oncol. 2006;79(2):224–30. 16697065 10.1016/j.radonc.2006.03.020

[14] Blanpain B and Mercier D . The delta envelope: a technique for dose distribution comparison. Med Phys. 2009;36(3):797–808. 19378740 10.1118/1.3070546

[15] Li H , Dong L , Zhang L , Yang JN , Gillin MT , Zhu XR . Toward a better understanding of the gamma index: investigation of parameters with a surface‐based distance method. Med Phys. 2011;38(12):6730–41. 22149855 10.1118/1.3659707 PMC3298565

[16] Ezzell GA , Burmeister JW , Dogan N , et al. IMRT commissioning: multiple institution planning and dosimetry comparisons, a report from AAPM Task Group 119. Med Phys. 2009;36(11):5359–73. 19994544 10.1118/1.3238104

[17] Nelms BE , Zhen H , Tome WA . Per‐beam, planar IMRT QA passing rates do not predict clinically relevant patient dose errors. Med Phys. 2011;38(2):1037–44. 21452741 10.1118/1.3544657 PMC3188652

[18] Zhen H , Nelms BE , Tome WA . Moving from gamma passing rates to patient DVH‐based QA metrics in pretreatment dose QA. Med Phys. 2011;38(10):5477–89. 21992366 10.1118/1.3633904

[19] Kruse JJ . On the insensitivity of single field planar dosimetry to IMRT inaccuracies. Med Phys. 2010;37(6):2516–24. 10.1118/1.3425781 20632563

[20] Carrasco P , Jornet N , Latorre A , Eudaldo T , Ruiz A , Ribas M . 3D DVH‐based metric analysis versus per‐beam planar analysis in IMRT pretreatment verification. Med Phys. 2012;39(8):5040–49. 22894429 10.1118/1.4736949

[21] Stasi M , Bresciani S , Miranti A , Maggio A , Sapino V , Gabriele P . Pretreatment patient‐specific IMRT quality assurance: a correlation study between gamma index and patient clinical dose volume histogram. Med Phys. 2012;39(12):7626–34. 23231310 10.1118/1.4767763

[22] Coleman L and Skourou C . Sensitivity of volumetric modulated arc therapy patient specific QA results to multileaf collimator errors and correlation to dose volume histogram based metrics. Med Phys. 2013;40(11):111715. 24320423 10.1118/1.4824433

[23] Heilemann G , Poppe B , Laub W . On the sensitivity of common gamma‐index evaluation methods to MLC misalignments in Rapidarc quality assurance. Med Phys. 2013;40(3):031702. 23464297 10.1118/1.4789580

[24] Nelms BE , Chan MF , Jarry G , et al. Evaluating IMRT and VMAT dose accuracy: practical examples of failure to detect systematic errors when applying a commonly used metric and action levels. Med Phys. 2013;40(11):111722. 24320430 10.1118/1.4826166 PMC8353583

[25] Gordon J and Siebers J . Addressing a gap in current IMRT quality assurance. Int J Radiat Oncol Biol Phys. 2013;87(1):20–21. 23664077 10.1016/j.ijrobp.2013.03.030

[26] Cadman PF . Comment on “IMRT commissioning: some causes for concern”. Med Phys. 2011;38(7):4464–65. 21859047 10.1118/1.3602464

[27] Ezzell G . Response to “Comment on ‘IMRT commissioning: some causes for concern’”. Med Phys. 2011;38(7):4466. 28525120 10.1118/1.3602506

[28] Molineu A , Hernandez N , Nguyen T , Ibbott G , Followill D . Credentialing results from IMRT irradiations of an anthropomorphic head and neck phantom. Med Phys. 2013;40(2):022101. 23387762 10.1118/1.4773309 PMC3555917

[29] Fraass B , Doppke K , Hunt M , et al. American Association of Physicists in Medicine Radiation Therapy Committee Task Group 53: quality assurance for clinical radiotherapy treatment planning. Med Phys. 1998;25(10):1773–829. 9800687 10.1118/1.598373

[30] Mijnheer B , Olszewska A , Fiorino C , et al. ESTRO booklet No. 7: Quality assurance of treatment planning systems: practical examples for non‐IMRT photon beams. Brussels: ESTRO; 2004.

[31] Van Dyk J , Barnett RB , Cygler JE , Shragge PC . Commissioning and quality assurance of treatment planning computers. Int J Radiat Oncol Biol Phys. 1993;26(2):261–73. 8491684 10.1016/0360-3016(93)90206-b

[32] Venselaar J , Welleweerd H , Mijnheer B . Tolerances for the accuracy of photon beam dose calculations of treatment planning systems. Radiother Oncol. 2001;60(2):191–201. 11439214 10.1016/s0167-8140(01)00377-2

[33] Palta JR , Kim S , Li J , Liu C . Tolerance limits and action levels for planning and delivery of IMRT. In: Palta JR, Mackie TR, editors. Intensity‐modulated radiation therapy: the state of art (Medical Physics Monograph No. 29). Madison, WI: Medical Physics; 2003 p.593–612.

[34] Gordon JD , Krafft SP , Jang S , Smith‐Raymond L , Stevie MY , Hamilton RJ . Confidence limit variation for a single IMRT system following the TG119 protocol. Med Phys. 2011;38(3):1641–48. 21520877 10.1118/1.3555298

[35] Almond PR , Biggs PJ , Coursey BM , et al. AAPM's TG‐51 protocol for clinical reference dosimetry of high‐energy photon and electron beams. Med Phys. 1999;26(9):1847–70. 10505874 10.1118/1.598691

[36] Howell RM , Scarboro SB , Kry SF , Yaldo DZ . Accuracy of out‐of‐field dose calculations by a commercial treatment planning system. Phys Med Biol. 2010;55(23):6999–7008. 21076191 10.1088/0031-9155/55/23/S03 PMC3152254

[37] Bednarz B and Xu XG . Monte Carlo modeling of a 6 and 18 MV Varian Clinac medical accelerator for in‐field and out‐of‐field dose calculations: development and validation. Phys Med Biol. 2009;54(4):N43–N57. 19141879 10.1088/0031-9155/54/4/N01 PMC3376900

[38] Huang J Y , Followill DS , Wang XA , Kry SF . Accuracy and sources of error of out‐of field dose calculations by a commercial treatment planning system for intensity‐modulated radiation therapy treatments. J Appl Clin Med Phys. 2013;14(2):4139. 23470942 10.1120/jacmp.v14i2.4139 PMC5714363

[39] Joosten A , Bochud F , Baechler S , Levi F , Mirimanoff R‐O , Moeckli R . Variability of a peripheral dose among various linac geometries for second cancer risk assessment. Phys Med Biol. 2011;56(16):5131–51. 21775792 10.1088/0031-9155/56/16/004

